# Load-Carrying Capacity of Thin-Walled Composite Columns with Rectangular Cross-Section under Axial Compression

**DOI:** 10.3390/ma17071615

**Published:** 2024-04-01

**Authors:** Patryk Rozylo, Michal Rogala, Jakub Pasnik

**Affiliations:** Department of Machine Design and Mechatronics, Faculty of Mechanical Engineering, Lublin University of Technology, Nadbystrzycka 36, 20-618 Lublin, Poland; m.rogala@pollub.pl (M.R.); j.pasnik@pollub.pl (J.P.)

**Keywords:** failure, closed profile, experimental study, FEM, axial compression

## Abstract

The aim of the current study was to determine the load capacity of composite columns subjected to axial compressive load. The subjects of the study were two types of columns with a rectangular cross-section, with different edge lengths. The tested columns had a closed cross-section. Four different fiber arrangements were analyzed for both cross-sections studied. The research was realized using interdisciplinary methods to determine the mechanism of damage to the composite material, with particular emphasis on damage initiation and propagation. Experimental tests were realized on a testing machine, the analysis was carried out with an acoustic emission system, and image analysis using visual assessment system of deflections of the walls of the structure. In addition, a number of numerical analyses were realized based on advanced modeling techniques for fiber-reinforced composites. A comparative analysis of both quantitative and qualitative results is presented for both analyses. The innovation of the presented research lies in the development of a custom method for modeling structures made of composite material with special emphasis on the failure phase. This will allow to accurately reflect the modeling of thin-walled structures with closed cross-section subjected to loading in a complex stress state.

## 1. Introduction

Composite structures with thin walls are a specific class of load-bearing systems that are extensively applied in numerous areas, such as aerospace, construction, and automotive. In general, carbon-fiber-reinforced polymer composite (CFRP) [1,2] or glass-fiber-reinforced polymer composite (GFRP) [3,4] materials are often used as load-carrying capacity profiles in the aerospace as well as construction industries. These structures can be designed in open- [5,6,7] as well as closed-section structures [8,9,10]. Research on closed-section composite profiles, which are widely used as load-carrying or stiffening components in structural systems, is the subject of current investigation. While open-section profiles with thin-walled composites have been widely used in the aforementioned industries, closed-section profiles offer improved strength and stiffness conditions. Composite materials exhibit unique behaviors when subjected to compressive loading, which is commonly referred to as buckling [11,12]. This phenomenon (buckling) is characterized by a change in the structure’s form due to compressive loading [13,14]. Initially, the walls of the composite structure are only in compression (pre-buckling condition), after which further loading of the structure causes the buckling phenomenon and the achievement of critical load. After the buckling state is reached, it is important during further loading of the structure that the post-buckling equilibrium path demonstrate a stable character (i.e., that the increase in deflection is accompanied by an increase in load) [15]. The buckling problem has been the subject of scientific interest of many researchers [16,17,18]. For thin-walled profiles made of composites, it is possible to reach failure loads that exceed critical loads several times [19,20]. The arrangement of the laminate layers can be chosen to increase or decrease the stiffness of the structure. Therefore, the critical load or load-carrying capacity of the structure depends on the careful selection of the layer arrangement in the composite material [21,22,23]. Critical loads for experimental studies are typically based on well-known approximation techniques, as shown in numerous scientific papers [24,25]. In numerical calculations based on the finite element (FE) method, determining the critical state involves solving a linear eigenproblem, which is based on the minimum potential energy criterion of the system [19,26]. 

While conducting advanced studies on the stability and load-carrying capacity of composite profiles, it is crucial to assess the structure’s behavior in the buckling state accurately. However, it is equally important to determine the structure’s behavior in the post-buckling condition, especially during the damage phase of the material. During experimental studies, it is crucial to determine the loads at which initiation of damage occurs, which often involves damage to the first ply of the composite material [27] or the first signs of delamination [28,29,30]. With further loading of the structure, the damage increases, and subsequent layers of the composite material are damaged, causing the loss of the structure’s load-carrying capacity (i.e., the structure’s inability to continue to carry the compressive load). An in-depth analysis of the behavior of composite materials can help to understand the complex failure mechanism. Research is typically conducted using research methods that enable the analysis of a structure’s various stages of failure, from the initial damage phase known as initiation of damage to complete failure. Regarding the experimental study, a range of methods is typically used to analyze the post-buckling behavior, such as universal testing machines, acoustic emission methods, and systems that allow optical measurement of structural deformation. These methods, such as Aramis, which allows for optical measurement of deformation, can assess the structure’s damage quantitatively and qualitatively. Regarding the numerical analysis using the (Finite Element Method) FEM, nonlinear simulations are performed to assess the post-buckling behavior, taking into account the damage models of the composite material. This allows the correct identification of the initiation of damage of the composite material using criteria and advanced damage models. The use of numerical simulations provides an assessment of the level of fiber and matrix damage to the composite material in compression or tension and the contribution of layered shear through progressive failure analysis (PFA). Additionally, it is possible to assess failure state in the context of delamination using cohesive zone model (CZM) [31,32,33,34], or due to cracking of the composite material on the basis of the extended finite element method (XFEM) [35,36]. 

The research presented in this article employs a combination of multiple testing techniques and numerical simulations to carefully evaluate the stability and load-carrying capacity of thin-walled composite structures under axial compression. Evaluating the results of critical and boundary conditions tests, the researchers can accurately describe the behavior of thin composite structures subjected to axial loading and comprehensively analyze their failure mechanisms. The study focuses on assessing the initiation and progression of the damage mechanism of composite structures with square cross-sections subjected to compression. The research was conducted under the National Science Centre project (Poland)—number 2021/41/B/ST8/00148. The studies conducted in this article are a follow-up and an extension of the research presented in the papers [37,38].

One of the novel aspects of this paper is the authors’ development of numerical models to describe the failure mechanism of composite materials, confirmed by the acoustic emission technique and deformation measurement results. Another aspect is the closed cross-section of the thin-walled profile. Careful study of the failure mechanism of the structure will allow for assessing its ability to carry loads in a combined load state and compare it with the results for columns with an open cross-section. The method of evaluating the failure phenomenon was based on the use of an advanced nonlinear numerical model based on progressive failure analysis (PFA)—which made it possible to assess the damage initiation (Hashin criterion) and the further damage evolution up to failure (energy criterion). Parallel analysis of the failure of composite structures using the acoustic emission (AE) method and the universal testing machine (UTM) allowed the evaluation of the complex mechanism of failure. More details of similar studies are presented in the paper [38].

## 2. The Subject of the Research

**Figure 1:**
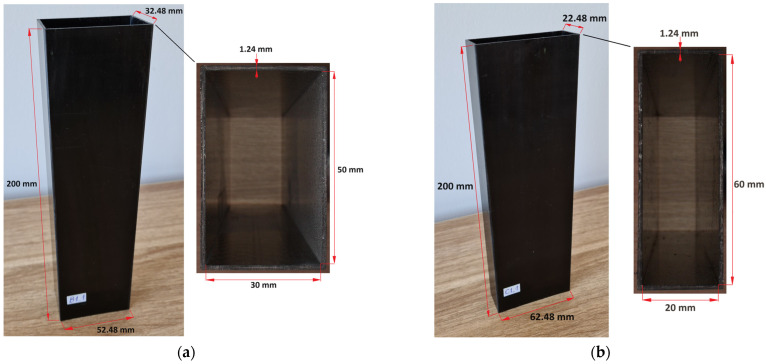
Specimen: (**a**) B-type, (**b**) C-type.

The subject of the following article was closed-section columns made of carbon–epoxy composite. The thin-walled columns were each composed of 8 layers that had different fiber orientations. Four different stacking sequences were used in the study, labeled with consecutive numbers, from 1 to 4. These were: B1—[0°/45°/−45°/90°]_s_, B2—[0°/90°/0°/90°]_s_, B3—[45°/−45°/90°/0°]_s_, B4—[90°/−45°/45°/0°]_s_. Similarly, the same arrangements were applied to the section labeled C and were respectively referred to as: C1, C2, C3, and C4. The labels B and C both relate to the cross-section types of the thin-walled column. Both cross-sections were rectangular in shape. The dimensions of cross-section B: 30 × 50 mm and 1.24 mm in thickness; while for cross-section C, the dimensions were 20 × 60 mm and 1.24 mm in thickness. For both profiles, the sizes were measured inside the profile, which gives 32.48 × 52.48 mm and 22.48 × 62.48 mm total size, respectively. For each configuration of fibers, three test specimens were experimentally tested, which were denoted as follows: B1_1, B1_2, B1_3, and accordingly for the other layouts and cross-sections. Consequently, the total number of profiles tested was 24 (three specimens with four layouts for both profiles, B and C). The composite columns were made by autoclave technology using prepreg strips designated as CYCOM 985-42%-HS-135-305 (Solvay, Tempe, AZ, USA). The designation 985 refers to the used resin system. The volume proportion of resin in the prefabricated material was 42%. The prepregs were reinforced with high-strength (HS) carbon fibers with a density of 135 g/m^2^, while the width of the base prepreg strip was 305 mm. Autoclave process parameters for the manufacturing of profiles were as follows: the autoclave curing temperature was 177 °C, while the overpressure was 0.6 MPa. The manufacture of the test specimens was outsourced to a company with extensive experience in the production of laminated thin-walled composite structures. This resulted in profiles with very-high-dimensional precision. Initially, trial specimens were fabricated and thoroughly inspected for dimensional accuracy and manufacturing errors typical for composite structures. This was done using a Keyence VHX 970F optical microscope (Keyence, Mechelen, Belgium). Composite columns are shown in Figure 1.

## 3. Experimental Research

The research included basic tests on a Zwick Z100 universal testing machine (ZwickRoell GmbH & Co. KG, Ulm, Germany) to determine load-carrying capacity [31]. Axial compression tests were performed on thin-walled composite columns at room temperature with constant velocity of crosshead movement rate of 1 mm/min. The buckling phase was identified through the observation of the buckling shape of the construction, and approximation methods were used to determine the critical loads [15,23]. Studies of this stage have been included in previous publications, which described profiles with square [38] and rectangular [37] cross-sections, respectively.

**Figure 2:**
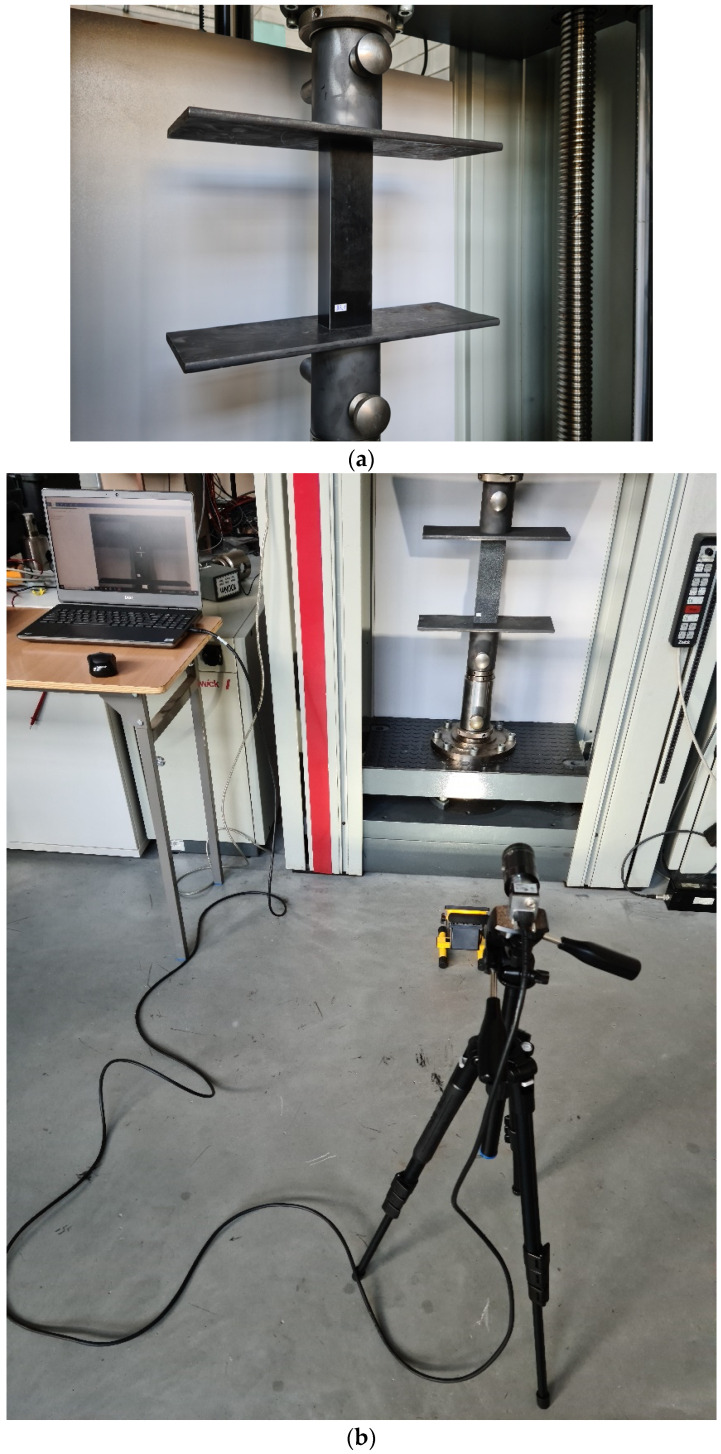
Experimental test stand: (**a**) test specimen with testing machine heads, (**b**) general view of universal testing machine Zwick Z100 and Aramis 2D system.

**Figure 3:**
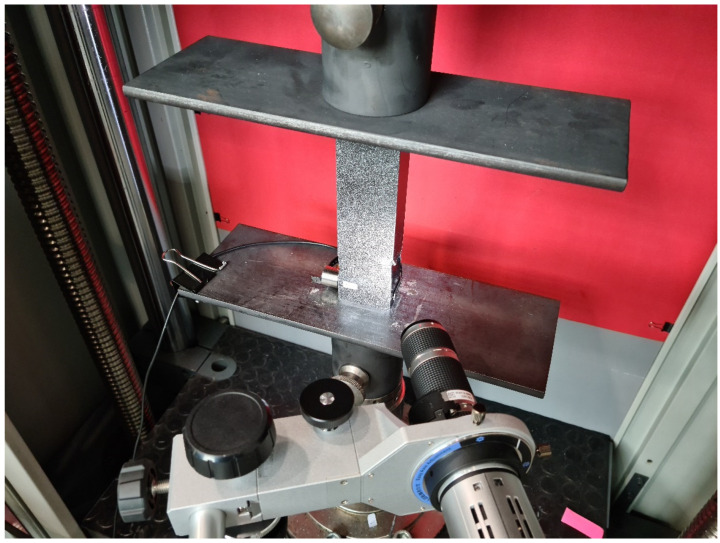
Experimental test stand and Keyence VHX-970F microscope.

The primary goal of the experimental investigation was to identify the post-buckling condition or the paths that were identified throughout the full load range and up until the structure failed. As stated in the previous section on the study’s subject, tests were conducted on a total of 24 real specimens (each with a distinct composite material layer) using a universal testing machine. Furthermore, an optical deformation measurement system called the ARAMIS 2D (Carl Zeiss GOM, Braunschweig, Germany) digital image system [39,40] was used in the stand tests. The use of the described system allows for recording the deformation of composite structures, especially the failure phase. A graphical illustration of the test stand is given in Figure 2. Special experimental heads with flat, parallel working surfaces were used for axial compression tests. These heads were rigidly fixed within the top crosshead and bottom of the testing apparatus. Figure 3 presents a graphical representation of the experimental testing heads fixed in the testing apparatus.

Furthermore, the AMSY-5 (Vallen Systeme GmbH, Icking, Germany) acoustic emission signal (AE) measuring device [30,41] was used in tests. This system, which allows for registration of AE signals, includes the assessment of failure to structures made of composites. 

In the physical study, a non-reflective (mats) background was used to provide precise measurement of the deflection of the column using the Aramis system. However, the strong brightness of the specimen caused unwanted overexposure of the columns, which adversely affected the recording of deformations. To solve this problem, a filter was used to absorb the unwanted effects of overexposure. It was necessary to use LED lamps to ensure adequate brightness of the exam samples for ongoing evaluation of the sequence of captured images and proper evaluation of the structure’s under-compression load.

The study also involved a digital microscope Keyence—VHX-7000 series, model VHX-970F (Keyence, Mechelen, Belgium), which included a VHX-A97FP control console, VHX-H5M 3D measurement software, VHX-7020 high-performance camera, VH-Z20T zoom lens (magnification 20×–200×), VH-Z00T zoom lens (magnification 0×–50×) and VHX-S600E tripod. The microscope had a special movable head that made it possible to record forms of structural damage (Figure 3). The research employed an articulated arm that was attached to the movable head of the microscope, enabling precise observation of the composite material’s failure.

The study’s objective was to determine the damage initiation and the failure load. The abovementioned parameters were determined using the approximation technique and by analyzing certain acoustic emission signals after the composite material had reached its post-buckling equilibrium path. Additionally, the study observed the modes of buckling and damage of the column structure. The research was focused on the axial compression of the profiles and relied on the goals of the investigations from the National Science Centre project. Each specimen was comprised of three samples on each stacking sequence. The analyzed material properties shown in Table 1 were the result of the company’s own experimental studies, and their use in the FEM simulations allows to determine the contribution of the fibers and matrix to the progression of the failure mechanism of the structure. The material properties used in the numerical analyses were determined as defined in Section 2 and were restricted to certain mechanical properties necessary for the modeling object of the research.

## 4. Numerical Simulation

Numerical simulations for the loading of composite columns were conducted in the Abaqus software (Abaqus 2023, Dassault Systemes Simulia Corporation, Velizy Villacoublay, France). A dedicated material model was used for this purpose, which allows assigning material parameters, which were obtained by the authors in the basic research described above, for each layer. Parameters characterizing material failure were derived from the literature [42,43]. In order to correctly replicate the behavior of the profile during the compression test, numerical simulations using FEM were performed in two steps. The first stage consisted of determining the form of buckling (linear stability—buckling problem) and the corresponding value of the critical force. The numerical problem described above involves solving an eigenproblem on the basis of the minimum potential energy criterion. The outcome of this operation is to determine the buckling form of a thin-walled composite column. The results of the work within the first stage described above are thoroughly explained by the authors in the paper [37]. By obtaining the critical force and the buckling mode, one could determine the critical state of the structure.

The second stage of the numerical analysis was nonlinear FEM simulations of the stability problem. The results of the previous stage of the analysis were used as input (the form of the buckling) in the next step. In addition, a geometric imperfection with an amplitude of *w*_0_ = 0.05 mm (of the profiles wall thickness) was introduced. The value of the initial deflection was defined on the basis of the preliminary numerical analyses carried out previously, in which it was verified under which value of the deflection the results of both the numerical analysis and the experimental test are comparable. The nonlinear stability simulations were performed using an incremental–iterative Newton–Raphson method. The technique used provided an analysis of the structure under compressive loading of thin-walled composite profiles. In addition, an advanced material failure model—Progressive Failure Analysis (PFA)—was used. As stated in the Abaqus documentation, PFA is based on the equations given by Hashin (damage initiation criterion) [44,45]. The equations below describe the conditions required to achieve composite failure by fiber tension (1), fiber compression (2), matrix compression (3), and matrix tension (4):

(1) $$F_{f}^{t}={\left(\frac{{\hat{\sigma }}_{11}}{X^{T}}\right)}^{2}+\alpha {\left(\frac{{\hat{\tau }}_{12}}{S^{L}}\right)}^{2}=1,\;where\;\left({\hat{\sigma }}_{11}\geq 0\right)$$

(2) $$F_{f}^{c}={\left(\frac{{\hat{\sigma }}_{11}}{X^{C}}\right)}^{2}=1,\;where\;\left({\hat{\sigma }}_{11}<0\right)$$

(3) $$F_{m}^{t}={\left(\frac{{\hat{\sigma }}_{22}}{Y^{T}}\right)}^{2}+{\left(\frac{{\hat{\tau }}_{12}}{S^{L}}\right)}^{2}=1,\;where\;\left({\hat{\sigma }}_{22}\geq 0\right)$$

(4) $$F_{m}^{c}={\left(\frac{{\hat{\sigma }}_{11}}{2S^{T}}\right)}^{2}+\left[{\left(\frac{Y^{C}}{2S^{T}}\right)}^{2}-1\right]\frac{{\hat{\sigma }}_{22}}{Y^{C}}+{\left(\frac{{\hat{\tau }}_{12}}{S^{L}}\right)}^{2}=1,\;where\;\left({\hat{\sigma }}_{22}<0\right)$$

where *X^T^*, *X^C^* stand for tensile/compressive strength in longitudinal direction, *Y^T^*, *Y^C^* for tensile/compressive strength in transverse direction, and *S^L^*, *S^T^* for shear strength in longitudinal/transverse direction, respectively. *α* is the contribution of the shear stress (to the fiber tensile initiation criterion), ${\hat{\sigma }}_{11},{\hat{\sigma }}_{22},{\hat{\tau }}_{12}$ are the components of the effective stress tensor (5).

Damage initiation occurs when any of Equations (1)–(4) are satisfied, i.e., any component of initiation of damage criterion achieves a value of 1. The most relevant approach to numerical analysis from the point of view of damage initiation analysis is the approach in which damage is defined as the loss of effective cross-section area [46]. In view of the above, the *d* coefficient has been proposed as a scalar damage coefficient to describe the damage phenomenon. When the *d* coefficient reaches a value of 0, there is no damage. When the *d* coefficient achieves a value of 1, damage occurs. In addition, the definition of effective stress is as follows [47]:

(5) $$\hat{\sigma }=\frac{1}{1-d}\sigma =M\sigma =\left[\begin{array}{ccc} \frac{1}{1-d_{f}} & 0 & 0\\ 0 & \frac{1}{1-d_{m}} & 0\\ 0 & 0 & \frac{1}{1-d_{s}} \end{array}\right]\left\{\begin{array}{c} \sigma_{11}\\ \sigma_{22}\\ \tau_{12} \end{array}\right\}$$

where σ is the apparent (Cauchy nominal) stress; M denotes the damage operator; $\hat{\sigma }$ constitutes the effective stress; *d* is that aforementioned damage parameter; *d*_f_, *d*_m_, and *d*_s_ refer to the parameters defining damage modes to fiber, matrix and shear; $\sigma_{ij}$ are the stresses on the appropriate directions.

Damage to an anisotropic material reinforced with long fibers is manifested by a degradation of the material’s stiffness matrix coefficients. Such a model, implemented directly in Abaqus, was used in this case. Based on Equation (5) and a quantitative evaluation of the Poisson’s ratio degradation [48], the compliance matrix including damage can be represented as follows:

(6) $$F=\left[\begin{array}{ccc} \frac{1}{(1-d_{f})E_{1}} & -\frac{\nu_{21}}{E_{2}} & 0\\ -\frac{\nu_{12}}{E_{1}} & \frac{1}{(1-d_{m})E_{2}} & 0\\ 0 & 0 & \frac{1}{(1-d_{s})G_{12}} \end{array}\right]$$

On the other hand, the corresponding damaged (elasticity) matrix can be shown as

(7) $$C=\left[\begin{array}{ccc} (1-d_{f})E_{1} & (1-d_{f})(1-d_{m})\nu_{21}E_{1} & 0\\ (1-d_{f})(1-d_{m})\nu_{12}E_{2} & (1-d_{m})E_{2} & 0\\ 0 & 0 & A(1-d_{s})G_{12} \end{array}\right]$$

The parameter *A* in Formula (7) represents:

(8) $$A=1-\nu_{12}\nu_{21}(1-d_{f})(1-d_{m})$$

All of the parameters presented above are described in detail in [38]. Once damage initiation is satisfied, further loading of this thin-walled profile will degrade the material stiffness parameters. Damage variables control this process. The energy of fracture *Gc* released during failure propagation is the basis for damage evolution. Damage evolution starts at the moment when the initiation of damage condition based on the Hashin criterion is fulfilled. By doing so, it is possible to perform progressive damage analysis (PFA), which has been described in detail in the article [38].

Once an initiation has taken place, the damage parameter for a given mode will be represented in the following way:

(9) $$d=\frac{\delta_{eq}^{f}\left(\delta_{eq}-\delta_{eq}^{0}\right)}{\delta_{eq}\left(\delta_{eq}^{f}-\delta_{eq}^{0}\right)}$$

where $\delta_{eq}^{0}$ denotes the initial (equivalent) displacement at which the damage initiation criterion is met; $\delta_{eq}^{f}$ denotes the displacement whereby the material is fully damaged. The parameter that must be determined for damage evolution analysis, in addition to displacement, is the amount of energy dissipated during damage propagation. When considering damage evolution, four energy parameters are required. These are: energy dissipated during fiber tension $G_{ft}^{c}$ and compression $G_{fc}^{c}$; $G_{mt}^{c}$ and $G_{mc}^{c}$ stand for the amount of energy dissipated due to matrix tension and compression, respectively. The parameters describing the damage evolution in Abaqus software are as follows: damage evolution caused by fiber tension—DAMAGEFT; fiber compression—DAMAGEFC; matrix stretching is described as DAMAGEMT and matrix compression as DAMAGEMC. The last parameter is DAMAGESHR, which describes shear damage. In this sense, a detailed analysis of the damage evolution in the composite material, including the load-carrying capacity, is possible.

The main objective of establishing the FEM model was to reproduce the actual model as closely as possible. The specimen on the basis of which the model was created was a thin-walled composite profile, consisting of 8 plies of carbon–epoxy composite material. Each layer was of equal thickness. Loading and boundary conditions were implemented using two identical non-deformable plates. The analyzed model was a composite column with a closed rectangular cross-section. Two separate FEM models were created for each of the profiles, B and C, with the corresponding parameters and dimensions given in the previous paragraphs. These models were each prepared with four variants of fiber layouts. These layouts corresponded to those used in the experimental specimens and are as follows: B1—[0°/45°/−45°/90°]_s_, B2—[0°/90°/0°/90°]_s_, B3—[45°/−45°/90°/0°]_s_, and B4—[90°/−45°/45°/0°]_s_ and, consequently, C1—[0°/45°/−45°/90°]_s_, C2—[0°/90°/0°/90°]_s_, C3—[45°/−45°/90°/0°]_s_, and C4—[90°/−45°/45°/0°]_s_. 

**Figure 4:**
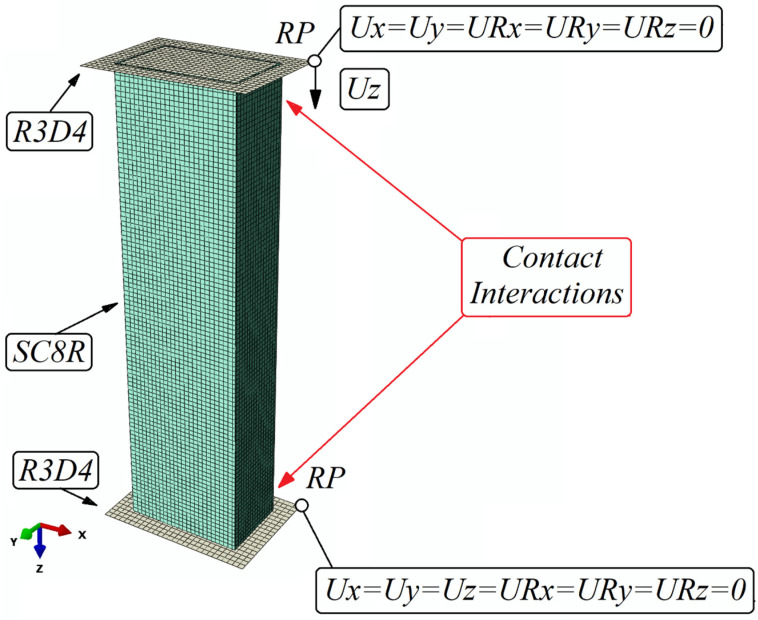
Numerical model of thin-walled structure.

The numerical model was prepared based on continuum shell elements with maintaining a physical representation of the profile wall thickness. The composite column consisted of 8 plies of composite, while the boundary conditions (BC) were assigned to non-deformable flat plates created as shell elements. For the column material, eight-node quadrilateral in-plane general-purpose continuum shell elements having only three translational degrees of freedom at the node were selected with reduced integration and hourglass control. For the non-deformable plates, 4-node three-dimensional rigid quadrilateral elements with 6 degrees of freedom per node were assigned. The composite column was meshed with a specified mesh density of 2 mm, while the non-deformable plates had a mesh with a density of 2.5 mm assigned. The numerical model consisted of 10,320 finite elements, including 9200 SC8R-type hexahedral elements and 1120 R3D4-type linear quadrilateral elements. The total number of nodes in the model was 19,802. A contact relationship was introduced between the plates and the column to simulate the actual interaction of the elements in the tangential and normal directions. Friction between the composite column and the non-deformable plates was included in the model with a coefficient of 0.2. Boundary conditions were defined at a specially selected reference point and connected to the non-deformable plates. For the point on the bottom plate, all degrees of freedom were locked to prevent any movement of the support. For the top plate, the reference point was withdrawn from all degrees of freedom except one, movement in the *Z*-axis direction. This allowed the load to be realized by compressing the specimen at this point. The load was implemented by assigning an assumed displacement to the top plate in the *Z*-axis direction. The boundary conditions, the load, the types of finite elements assigned, and the pictorial view of the B-section numerical model in Abaqus are shown in Figure 4. The parameters presented below are the analogue of those for the section C profile.

The finite element mesh size used in the model was not chosen arbitrarily. A thorough analysis of the influence of mesh size on the accuracy and compatibility of numerical analysis results with experimental data was performed. The study showed that a mesh size of 2 mm produced the most similar analysis results to those obtained from experimental studies. Exemplary analysis of the influence of mesh size on the accuracy of the obtained results was presented for closed-section profiles in the paper [37].

Numerical simulations using the FEM were performed using widely known and commonly used computational methods and material failure models. The damage initiation was based on the Hashin criterion, while further evolution of damage was based on the energy criterion. In addition, today’s widely used continuum shell element models allow a detailed analysis of the failure of the composite structure to determine the exact location of the damage as well as the type of material damage—whether it is failure due to tension or compression of the fibers, or compression or tension of the matrix or shear damage between the layers. Future studies will be designed based on the cohesive element method, which will make it possible to analyze the structure for the presence of delamination that has been observed in experimental investigations.

## 5. Research Results

The use of experimental methods as well as numerical simulations with the FEM enabled the assessment of damage initiation and propagation in thin-walled composite columns. Through the utilization of multidisciplinary testing techniques, the load-carrying capacity of the columns was determined. Additionally, advanced failure models of the composites were used to determine post-buckling states through numerical simulations.

**Figure 5:**
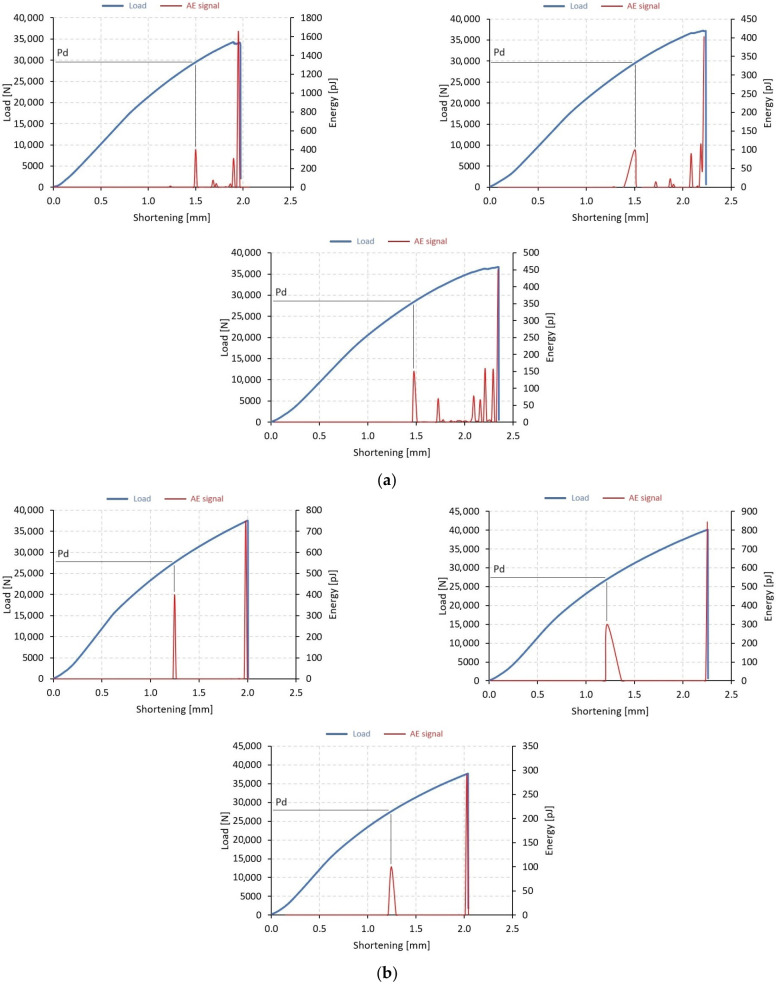

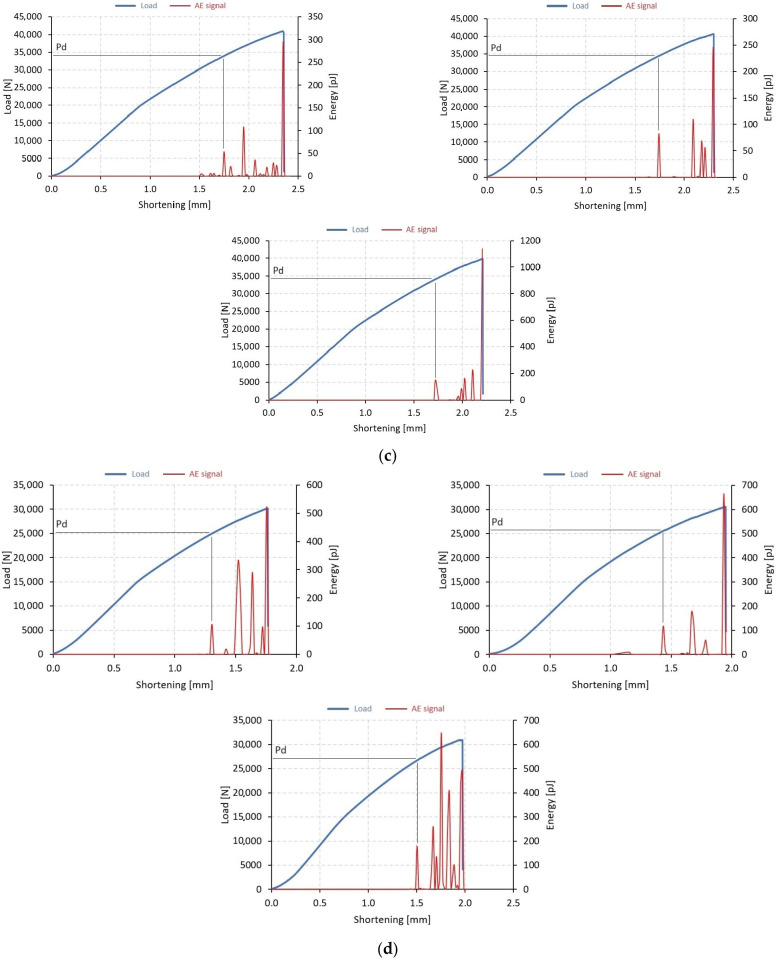
Post-buckling equilibrium paths with AE signals—an experimental study: (**a**) specimen B1 (B1_1, B1_2, B1_3), (**b**) specimen B2 (B2_1, B2_2, B2_3), (**c**) specimen B3 (B3_1, B3_2, B3_3), (**d**) specimen B4 (B4_1, B4_2, B4_3).

**Figure 6:**
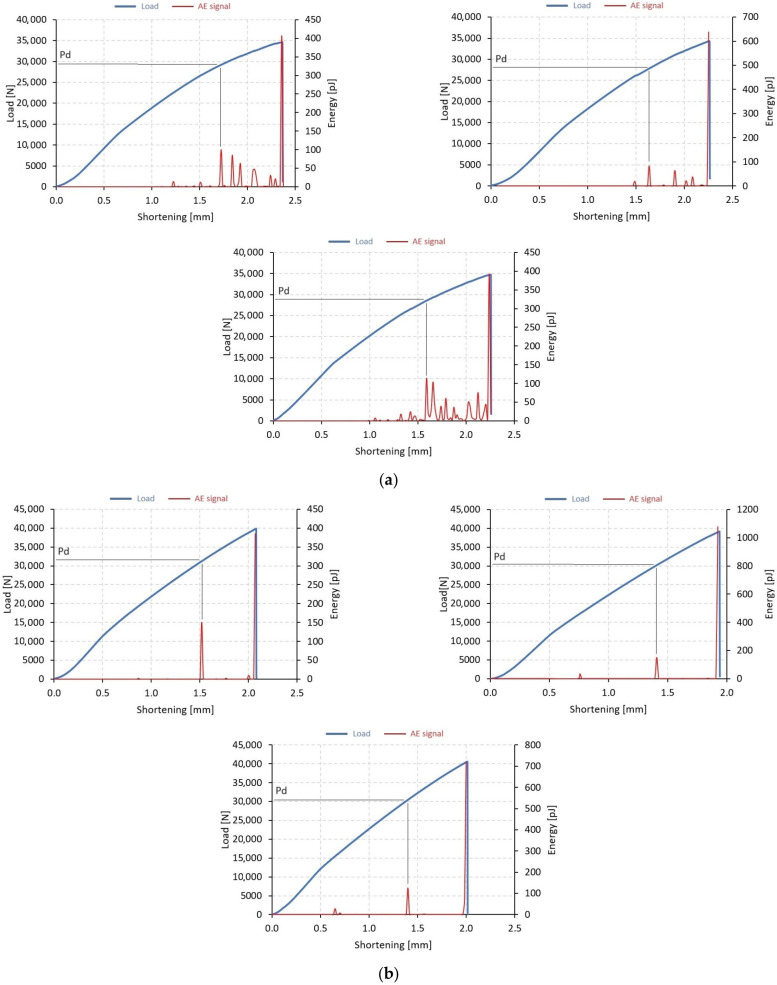

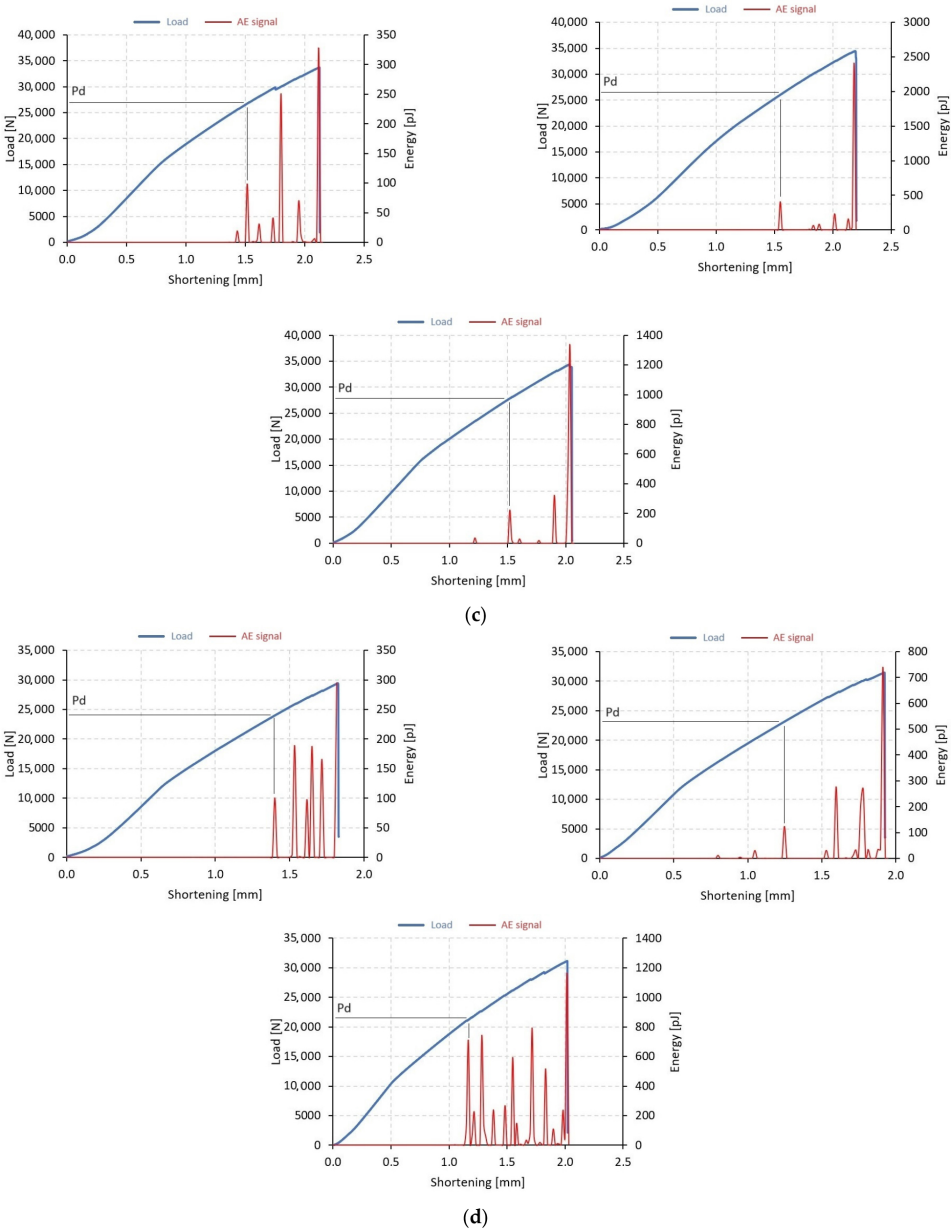
Post-buckling equilibrium paths with AE signals—an experimental study: (**a**) specimen C1 (C1_1, C1_2, C1_3), (**b**) specimen C2 (C2_1, C2_2, C2_3), (**c**) specimen C3 (C3_1, C3_2, C3_3), (**d**) specimen C4 (C4_1, C4_2, C4_3).

The evaluation of the post-buckling phase has enabled researchers to study the behavior of composite columns under a full range of loads. This analysis allows for the determination of the load-carrying capacity of composite columns, which is closely associated with damage initiation, progression, and total failure. To investigate the post-buckling behavior of thin-walled columns, various experimental methods were employed, including a universal testing machine, an AE method, Aramis system, as well as a digital microscope with a moving head to record damage form. The primary method used for quantitative evaluation of test results was post-buckling equilibrium paths, which were compared with AE signals, as demonstrated in Figure 5 and Figure 6, where the blue curve represents the load-shortening relationship, and the red one represents the selected AE signal (energy) dependence on shortening. By comparing the equilibrium paths with the AE signals, researchers were able to determine the loads causing the failure of the composite and the loads which cause the loss of load capacity. In this case, the AE signal was found to be more reliable for assessing the loads initiating damage.

The experimental stage determined the loads at which damage to the composite material was first recorded, and these are denoted as *P*_d_ in Figure 5 and Figure 6. To obtain a clearer picture, the average value (avg) of *P*_d_ was calculated from the testing of 3 specimens of each type, B1–B4 and C1–C4. For the failure loads that resulted in the loss of load-carrying capacity, the maximum load recorded in the equilibrium paths after buckling was used to determine the value of P_f_. Similarly, the average value (avg) of *P*_f_ was calculated from three test samples for each of the four composite systems. In Figure 5 and Figure 6, the blue line indicates the load curve, while the red line indicates the acoustic emission signal (energy signal).

The results shown below refer to two types of thin-walled profiles, B and C. In addition, each series sets three consecutive samples with the same fiber arrangement, e.g., Figure 5a. The tests showed that the acoustic emission signal accompanying the initiation of damage to the structure corresponded to a visible increase in the signal on the graph. The observed signal rise applied to both types of specimens analyzed, as well as the different fiber arrangements used.

**Figure 7:**
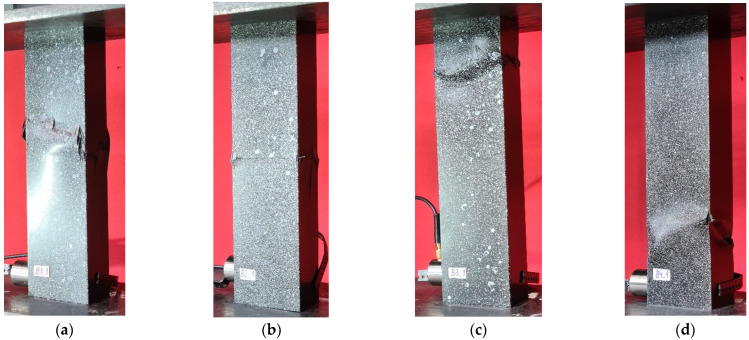

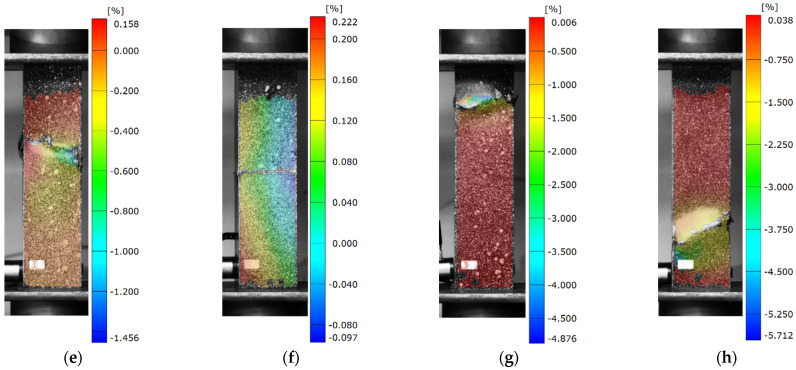
Failure of columns—experimental tests: (**a**,**e**) specimen type B1, (**b**,**f**) specimen type B2, (**c**,**g**) specimen type B3, (**d**,**h**) specimen type B4.

After conducting load-carrying tests, it was determined that the arrangement of the composite affected the damage initiation and failure loads. Specifically, the B3-type specimen with the arrangement [45°/−45°/90°/0°]_s_ had the highest value of damage initiation load as well as value of failure load. Interestingly, the different type specimen showed the highest reserve of load-bearing capacity, as the ratio of failure load to damage initiation load was B2 and C4 specimen, which correspond to [0°/90°/0°/90°]_s_ and [90°/−45°/45°/0°]_s_ composite layup, respectively. The bench tests conducted during the research consisted of a qualitative assessment of the findings. The results recorded during the study were expressed graphically, showing the damage profile from the experimental test stand (from the universal testing machine). The structural damage allowed for an accurate assessment of strength loss in a qualitative evaluation. It was noted that damage caused by the simultaneous destruction of composite material layers and delamination most commonly occurred in the middle part of the composite structure. In many cases, progressive delamination and cracking phenomena were visible, indicating a complex failure mechanism. The failure occurred in the region located near the center of the composite column’s height, regardless of the composite lay-ups. The results shown in Figure 7 represent the moment of loss of carrying capacity of thin-walled composite structures (B-type).

**Figure 8:**
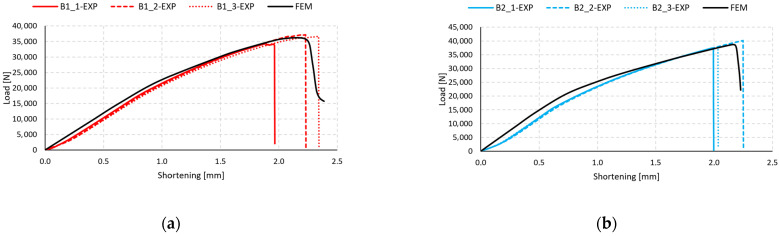

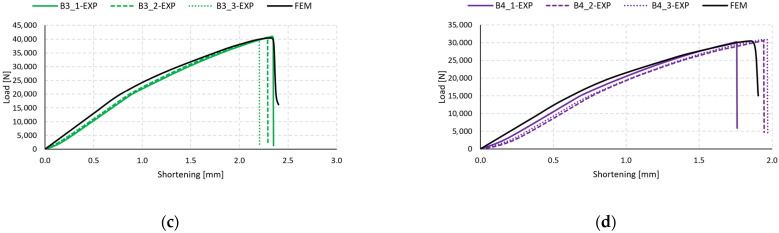
Comparison of numerical and experimental post-buckling equilibrium characteristics: (**a**) specimen type B1, (**b**) specimen type B2, (**c**) specimen type B3, (**d**) specimen type B4.

Equilibrium paths were determined in both numerical simulations and experimental studies, allowing for the determination of failure loads *P*_f_. The equilibrium paths determined in the simulation model are presented in Figure 8 (B-type).

It was found that the numerical simulations at the initial stage showed much higher “stiffness” than the experimental curves, which was caused by the fact that the FEM model was not affected by manufacturing imperfections, while the numerical models reflect an ideal structure without geometric disturbances. However, in each case, the tendency to match the numerical curve with the average of the three experimental curves persisted, confirming the correctness of the numerical models. Differences in the experimental characteristics were observed, especially in the case of shortening, where there was a loss of strength, which may have been caused by some imperfections during the manufacturing process of the composite columns.

**Figure 9:**
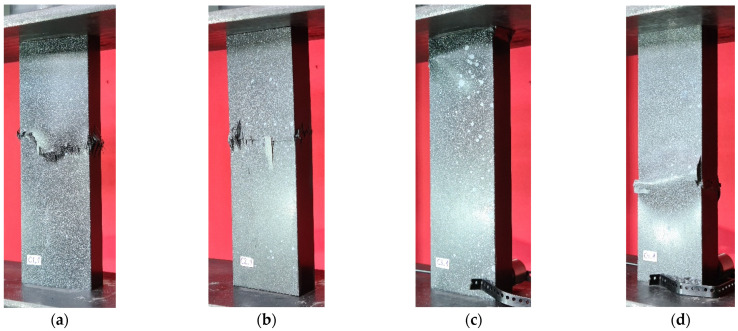

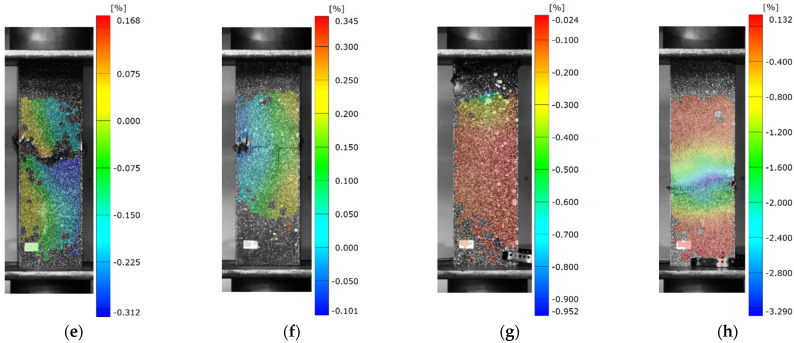
Failure of columns—experimental tests: (**a**,**e**) specimen type C1, (**b**,**f**) specimen type C2, (**c**,**g**) specimen type C3, (**d**,**h**) specimen type C4.

Stand tests of type C specimens mostly showed an analogous damage mechanism characterized by a clear fracture of the column in its central part. A slight movement of the damage area occurred in the case of specimen C3, as can be observed in Figure 9c. Interestingly, and worth noting, comparing the graphical presentation of columns of types B and C (Figure 7 and Figure 9), the movement of the damage area of the structure for the [45°/−45°/90°/0°]_s_ fiber arrangement is visible regardless of the dimensions of the cross-section. Similarly, for the other composite layouts, the visual assessment of the damage site shows a similar character. The results shown in Figure 9 represent the moment of loss of carrying capacity of thin-walled composite structures (C-type).

**Figure 10:**
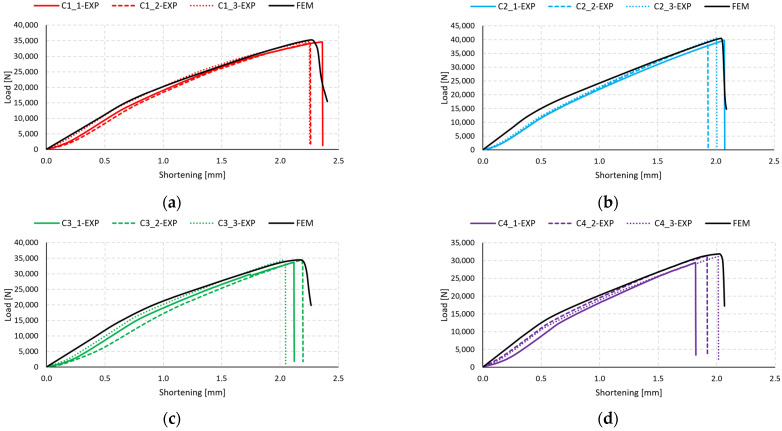
Comparison of numerical and experimental post-buckling equilibrium characteristics: (**a**) specimen type C1, (**b**) specimen type C2, (**c**) specimen type C3, (**d**) specimen type C4.

The present analysis provides a comparison of the equilibrium paths for type C specimens (Figure 10). In a manner similar to the plots for type B specimens displayed in Figure 8, the characteristics from FEM in the early stages exhibit a gentle increase in force relative to the experimental specimens. A slight discrepancy is evident, particularly for samples C1 and C2 (Figure 10a,b). For specimens C3 and C4, a similar pattern is observed in the initial stage of compression up to 0.7 mm. These discrepancies may be attributed to the inaccuracies present in the manufacturing process of the experimental samples. Unlike the thin-walled columns modeled using the finite element method, which did not account for geometric imprecisions, the FEM curves show “stiffer” characteristics at the initial stage of the structure’s operation. The equilibrium paths determined in the simulation model are presented in Figure 10 (C-type).

However, it is important to note that these differences do not significantly affect the obtained force values that cause the initiation of damage to the structure or loss of load-carrying capacity.

Table 2 summarizes the findings of the experimental tests for the profile indicated as B. Four fiber layout configurations were compared. Three composite specimens were tested in each case. Damage initiating load and failure load values were determined. The average initiating load and the average failure load were calculated for each specimen. The highest value of damage initiation force was obtained for specimen B3—[45°/−45°/90°/0°]_s_, similarly, the highest load-carrying capacity (failure) load was obtained for the same layer configuration, which were 34,040 N and 40,495 N, respectively. The next step was to determine the ratio of the failure load to the damage initiation load. The highest value of this parameter was obtained for the specimen with B2 [0°/90°/0°/90°]_s_ configuration—1.41. The value of the parameter for specimen B1 was 1.24, while for other specimens, B3 and B4, it was equal to 1.19.

Table 3 summarizes the experimental results for the C-section specimens. The values of the damage initiation and the failure load are given, similar to the previous case. The average values of the forces from the three tests were compared for each system. The highest value of damage initiation force was obtained for specimen C2 with the [0°/90°/0°/90°]_s_ configuration, with a value of 30,503 N. The highest failure load was also obtained for this specimen, with a value of 39,841 N. The lowest values of these forces were obtained for specimen C4, and they were 22,708 N and 30,679 N, respectively. After that, a comparison was made between the values of the forces *P*_f_/*P*_d_. The highest value of this parameter was obtained for specimen C4—1.35. Specimen C1 exhibits the lowest value of the parameter—1.21. The other specimens, C2 and C3, reached the value of the parameter 1.31 and 1.28, respectively.

**Figure 11:**
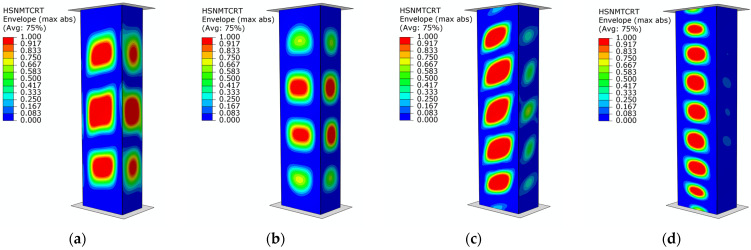
Damage initiation of columns—numerical study: (**a**) specimen type B1, (**b**) specimen type B2, (**c**) specimen type B3, (**d**) specimen type B4.

The results show good agreement between numerical simulations and experimental tests. In addition, in the results of the FEM simulations, damage initiation was evaluated based on the Hashin criterion. The values of the load initiating the damage of the composite structure are shown in Table 4. This criterion made it possible to determine the damage initiation loads (*P*_d_) as well as the areas where damage occurred, while determining whether fiber or matrix damage occurred and as a result of which type of loading. Accordingly, it was noted that *P*_d_ damage initiation occurred (Figure 11), respectively, for specimens of type B1—at a load of 30,028 N (as a result of matrix tension—HSNMTCRT), B2—at a load of 28,679 N (as a result of matrix tension—HSNMTCRT), B3—at a load of 36,018 N (as a result of matrix tension—HSNMTCRT), and B4—at a load of 25749 N (as a result of matrix tension—HSNMTCRT).

**Figure 12:**
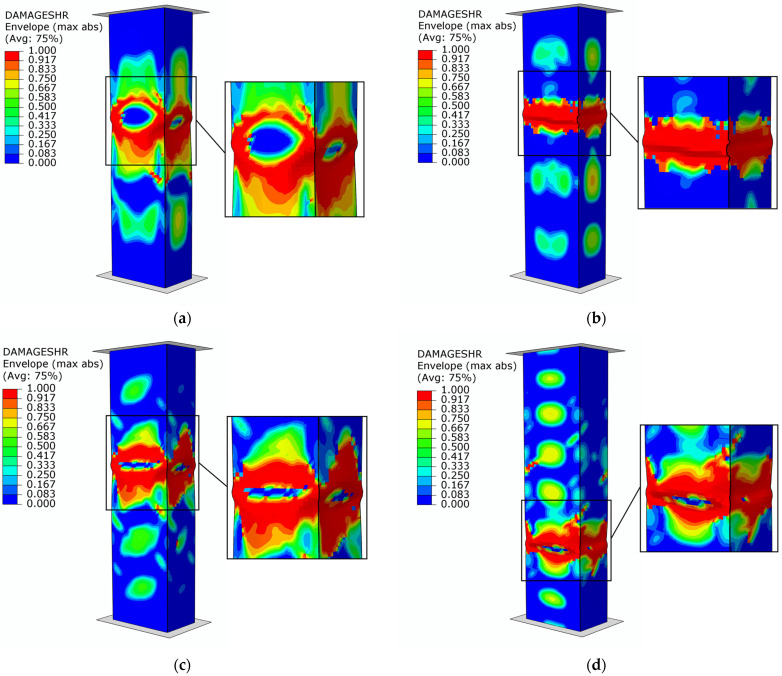
Failure of structures—numerical study: (**a**) specimen type B1, (**b**) specimen type B2, (**c**) specimen type B3, (**d**) specimen type B4.

The failure forms of type B structures are presented in Figure 12. The results of failure are described using a variable (DAMAGESHR parameter), as it is the most suitable for modeling with the FEM (it takes into account the results of the four parameters which are responsible for the evolution of damage, i.e., DAMAGEFC, DAMAGEFT, DAMAGEMC and DAMAGEMT) resulting from compression and tension of the fibers and matrix). The loss of the load-bearing capacity of the composite profiles occurred when a value of 1.0 was obtained for the parameter variable described above. For all types of samples (B1–B4), it was noted that the failure occurs due to structural failure in the center of the column. These results, at direct comparison of the graphical form with the physical specimens (Figure 7), confirm the quality of the conducted analyses, as evidenced by the obtained values of the forces specific to the damage initiation and failure presented in Table 2 and Table 4.

Table 4 presents the results of the numerical simulations. The table shows the values of initiation and failure loads for B-type profiles. The last column shows the *P*_f_/*P*_d_ parameter. The highest value, for both initiation and failure load, was obtained for specimen B3 with the following fiber layout—[45°/−45°/90°/0°]_s_. The lowest values of force, for both initiation and failure load, were obtained for specimen B4; they were the following values: initiation force 25,749 N and failure load 30,692 N. The ratio of *P*_f_/*P*_d_ was the highest for specimen B2, reaching 1.35, while the lowest for specimen B3 was 1.13. A similar trend can be seen in the experimental results, where the same specimens reached the highest and lowest values of the ratio, B2 and B3, respectively.

**Figure 13:**
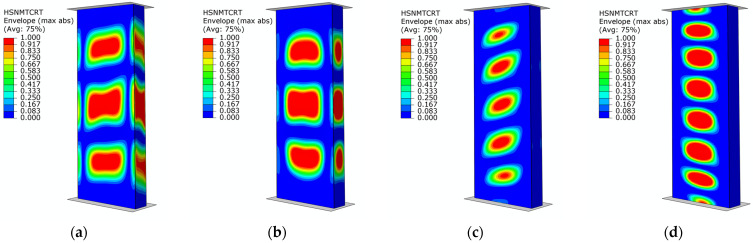
Damage initiation of structures—numerical simulations: (**a**) specimen type C1, (**b**) specimen type C2, (**c**) specimen type C3, (**d**) specimen type C4.

As in the case of B-type samples described above, the results of FEM simulations, damage initiation was evaluated based on the Hashin criterion. The damage initiation load (*P*_d_) of type C samples is shown in Table 5. Damage initiation *P*_d_ of the structure (Figure 13) was noted for type C1 specimens at a load of 28,978 N (as a result of matrix tension—HSNMTCRT), C2 specimens at a load of 31,074 N (as a result of matrix tension—HSNMTCRT), C3 specimens at a load of 27,103 N (as a result of matrix tension—HSNMTCRT), and C4 specimens at a load of 24,577 N (as a result of matrix tension—HSNMTCRT), respectively. It is worth noting that the highest value obtained for damage initiation (*P*_d_) occurred for B3- and C2-type profiles characterized by different fiber arrangements. Thus, it can be concluded that there is a relationship between the geometric dimensions of the cross-section, the fiber arrangement, and the value of the load causing damage initiation of thin-walled columns.

**Figure 14:**
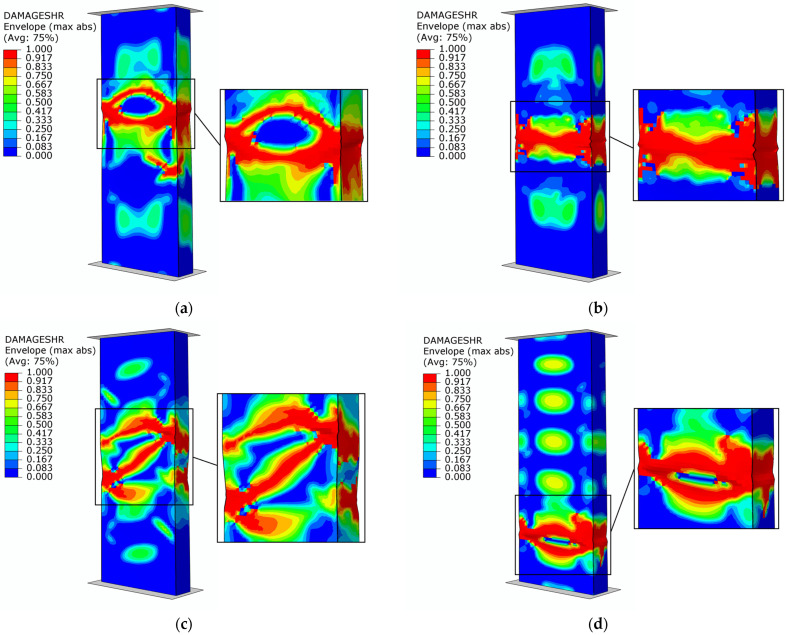
Failure of structures—numerical study: (**a**) specimen type C1, (**b**) specimen type C2, (**c**) specimen type C3, (**d**) specimen type C4.

The failure results of the numerical analyses for the C-type columns are shown in Figure 14. The identification of the damage mechanism was based on the output parameters detailed in the paragraph above Figure 12. The loss of load-carrying capacity occurred when the value of 1.0 was obtained for the DAMAGESHR parameter. The graphical form accompanying the failure is consistent with the experimental forms shown in Figure 9. In the case of the C3 specimen, a dislocation of the failure area was noted, which is due to geometric imperfections caused by the complex manufacturing process. This is because the numerical models did not contain the geometric inaccuracies with which the physical samples were burdened. However, despite the dislocation of the damage location, the equilibrium paths shown in Figure 10 confirm the high quality and convergence of the numerical analyses carried out with respect to stand tests.

The table below shows the values of damage initiation and failure loads for C1–C4 specimens. These values are the results of numerical simulations. The highest values of both initiating force and failure loads were obtained for the C2 specimen, while the lowest values were obtained for the C4 specimen. The *P*_f_/*P*_d_ parameter reached the highest value for specimens C2 and C4—1.30, while the lowest value for C1—1.22. The results of the FEM simulations were very similar to those of the experimental investigations, where the highest value of the parameter was obtained for specimen C4, while the lowest value was obtained for specimen C1, as in the numerical simulations. The same is true for the force values. In both cases, the highest values were obtained for specimen C2, while the lowest values were obtained for specimen C4.

Within the framework of the conducted research, it was observed that the damage of composite structures was initiated by a phenomenon known as damage initiation (which was associated with the failure of one of the layers of the composite material). During further loading of the structure, the phenomenon of damage evolution conditioned about further deepening of the damage to the composite material—up to the loss of the load-carrying capacity of the structure (inability to continue to carry compressive load). The obtained forms of damage proved that a complex mechanism of damage was obtained, where, in addition to local cracking of the layers of the composite material, local areas of delamination formation were observed. The above determines the necessity of further research using other advanced numerical damage models such as XFEM—allowing simulation of composite material cracking—and CZM—allowing simulation of delamination phenomenon. The research carried out in the study provides a high potential for practical application of the studied structures as load-bearing structures in actual structures. Due to the high strength properties and the complex mechanism of failure, the structures demonstrate very high compressive load strength [49].

## 6. Conclusions

The main focus of the present research was on two types of composite columns with different rectangular cross-sections. The tested columns were subjected to longitudinal compression testing, during which the damage initiation and the mechanism of its propagation until the loss of column strength were determined. The research was conducted on 24 physical samples consisting of two types of columns and four types of fiber bundles, each type of sample being produced and tested three times. The second stage of the research involved the development of proprietary numerical simulations reflecting the mechanical properties of the columns and their behavior throughout the entire range of loading. The numerical analysis was based on the finite element method using an advanced damage model based on the damage parameters, among which DAMAGESHR was the main parameter. Based on the conducted research, it was confirmed that the fiber bundle arrangement has a significant impact on the strength of the composite structure. The samples of types B and C showed variations in both the initiation and failure strength of the damage. The results of the conducted analyses showed high consistency in terms of both quality and quantity of data. The thin-walled structures showed a significant reserve of strength with respect to the onset of damage initiation, which was detailed in Table 2 and Table 3 for experimental studies and Table 4 and Table 5 for numerical analysis. Damage to the thin-walled structure occurred due to matrix tension in all numerical simulations. It is worth noting that all samples with the highest *P*_f_/*P*_d_ ratio for numerical analyses—B2 and C4, which correspond to [0°/90°/0°/90°]_s_ and [90°/−45°/45°/0°]_s_ composite layups, respectively—had the highest ratio based on the experimental data as well. Moreover, the lowest values of the ratio was observed for the same specimens both in experimental studies and numerical tests—B3 and C1. The high agreement between experimental results and the results of numerical analyses validates the accuracy of the established numerical models, and further research can be conducted using them. The assessment of the location of damage to the thin-walled structure showed that, in most cases, it occurs in the middle part of the column. The displacement of the location of damage to the structure occurred in the case of samples B3 and C3, which resulted from the assumption of ideal geometry of the column in the numerical analyses. The experimental samples are subjected to imperfections resulting from the complex production process of composite columns. The high convergence of the conducted analyses is also confirmed by the equilibrium paths shown in Figure 8 and Figure 10. The values of the forces associated with the initiation and loss of load capacity of the structure were calculated on the basis of the analysis of the acoustic emission signal.

## Figures and Tables

**Table 1:** Material properties of the CFRP material—average values (and standard deviation).

Mechanical Properties		Strength Parameters	
Young’s modulus *E*1 [MPa]	103,014.11 (2145.73)	Tensile Strength *F*TU (0°) [MPa]	1277.41 (56.23)
Young’s modulus *E*2 [MPa]	7361.45 (307.97)	Compressive Strength *F*CU (0°) [MPa]	572.44 (46.20)
Poisson’s ratio *v*12 [-]	0.37 (0.17)	Tensile Strength *F*TU (90°) [MPa]	31.46 (9.64)
Kirchhoff modulus *G*12 [MPa]	4040.53 (167.35)	Compressive Strength *F*CU (90°) [MPa]	104.04 (7.34)
-	-	Shear Strength *F*SU (45°) [MPa]	134.48 (2.71)

**Table 2:** Limit loads—experimental tests for B-type specimen.

Specimen Type	Specimen No.	*P*_d_ [kN]	*P*_d_ (avg) [N]	*P*_f_ [N]	*P*_f_ (avg) [N]	*P*_f_ (avg)/*P*_d_ (avg)
B1	B1_1	29,485	29,082	34,292	36,047	1.24
	B1_2	29,350		37,202		
	B1_3	28,409		36,647		
B2	B2_1	27,537	27,356	37,513	38,458	1.41
	B2_2	26,916		40,139		
	B2_3	27,615		37,722		
B3	B3_1	33,891	34,040	41,026	40,495	1.19
	B3_2	34,273		40,668		
	B3_3	33,957		39,790		
B4	B4_1	24,959	25,721	30,237	30,582	1.19
	B4_2	25,528		30,566		
	B4_3	26,676		30,945		

**Table 3:** Limit loads—experimental tests for C-type specimen.

Specimen Type	Specimen No.	*P*_d_ [N]	*P*_d_ (avg) [N]	*P*_f_ [N]	*P*_f_ (avg) [N]	*P*_f_ (avg)/*P*_d_ (avg)
C1	C1_1	29,183	28,473	34,615	34,551	1.21
	C1_2	27,742		34,315		
	C1_3	28,496		34,724		
C2	C2_1	31,081	30,504	39,800	39,841	1.31
	C2_2	30,128		39,151		
	C2_3	30,304		40,573		
C3	C3_1	26,653	26,758	33,662	34,148	1.28
	C3_2	25,897		34,417		
	C3_3	27,725		34,365		
C4	C4_1	23,901	22,708	29,449	30,679	1.35
	C4_2	23,064		31,416		
	C4_3	21,159		31,173		

**Table 4:** Limit loads—numerical simulations for B-type specimen.

Specimen	*P*_d_ [N]	*P*_f_ [N]	*P*_f_/*P*_d_
B1	30,028	36,207	1.21
B2	28,679	38,703	1.35
B3	36,018	40,624	1.13
B4	25,749	30,692	1.19

**Table 5:** Limit loads—numerical simulations for C-type specimen.

Specimen	*P*_d_ [N]	*P*_f_ [N]	*P*_f_/*P*_d_
C1	28,978	35,259	1.22
C2	31,074	40,463	1.30
C3	27,103	34,457	1.27
C4	24,577	31,885	1.30

## Data Availability

Data are contained within the article.
